# Multicellular Tumor Spheroids in Nanomedicine Research: A Perspective

**DOI:** 10.3389/fmedt.2022.909943

**Published:** 2022-06-15

**Authors:** Martina Rossi, Paolo Blasi

**Affiliations:** Department of Pharmacy and Biotechnology, University of Bologna, Bologna, Italy

**Keywords:** 3D *in vitro* models, cell culture monolayers, nanoparticles, drug delivery, drug targeting, high-throughput screening

## Abstract

Multicellular tumor spheroids are largely exploited in cancer research since they are more predictive than bi-dimensional cell cultures. Nanomedicine would benefit from the integration of this three-dimensional *in vitro* model in screening protocols. In this brief work, we discuss some of the issues that cancer nanomedicine will need to consider in the switch from bi-dimensional to three-dimensional multicellular tumor spheroid models.

## Prologue

Researchers in the drug delivery field routinely take advantage of *in vitro* cell culture models to assess the performance of nanotechnology-based medicines, with the aim of optimizing them prior to *in vivo* evaluations in animal models and predicting their performance in patients. This is of vital importance during the development of nanoparticles (NPs) or any other nanotechnology-based drug delivery system intended to target solid cancers. Today, most of the early-stage development of cancer nanomedicine products is done using cell culture monolayers, also termed bi-dimensional (2D) models. 2D cell cultures have been an essential tool in early-stage drug delivery investigations but they tend not to be useful for prediction, because they fail to replicate the tumor macrostructure, the tumor-stroma interaction, and the heterogeneity of the tumor microenvironment (1, 2). The three-dimensional (3D) architecture of human solid tumors provides optimal conditions for cellular organization, proliferation, and differentiation (3).

The need for more reliable and predictive *in vitro* models that respect the principle of the 3Rs (Replacing, Reducing, and Refining the use of *in vivo* experimentation) has motivated researchers to develop advanced and more consistent *in vitro* models. In this context, 3D cell cultures are rapidly taking the place of conventional 2D models in biomedical research. However, despite a consistent body of evidence suggesting that 2D models largely fail to predict NP performance and/or drug efficacy and efficiency *in vivo*, 3D models have not been fully integrated into nanomedicine research and are not routinely employed in screening procedures.

Among the *in vitro* 3D cancer models under scrutiny, multicellular tumor spheroids (MCTSs) have attracted great attention and are now broadly exploited in the cancer research field (4). Indeed, over the past decades, MCTSs has been used as models to study tumor cell metabolism, tumor growth and progression in a more realistic 3D context. Being relatively simple to produce and to grow, MCTSs have been regarded as the first choice to replace 2D cell cultures in the development of cancer nanomedicines (5).

In this brief work, we will discuss the use of MCTSs in drug delivery research, with a focus on cancer nanomedicine. After an overview of MCTS general features and preparation methodologies, we will discuss some issues to consider when switching from 2D to this 3D model, and we give our perspective on the potential of MCTSs in nanomedicine. Comprehensive reviews on the subject may be found elsewhere (6–8).

## Multicellular Tumor Spheroids

**Figure 1:**
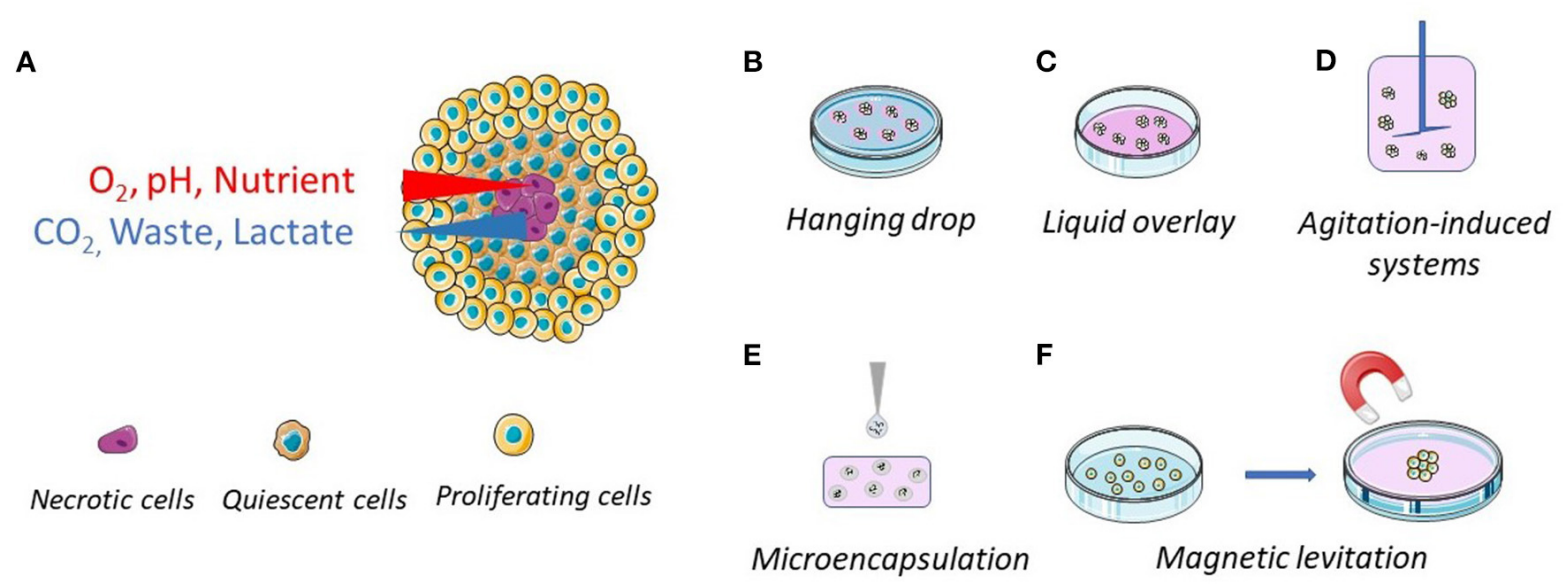
Schematic representations of the general structure of a multicellular tumor spheroid and of their common preparation methods. **(A)** The spheroid structure, with proliferating (outside layer), quiescent (intermediate layer) and necrotic (center) cells. The cellular density is lower in the outside layer. **(B)** Hanging drop method, where the cells come together at the bottom of a hanging drop. **(C)** Liquid overlay method, where the surface of plates or wells is coated with a non-adhesive material. **(D)** Spinner flasks (stirred or rotating vessel), where the cells are kept in suspension. **(E)** Microencapsulation and **(F)** magnetic levitation, where the cells, engulfed with magnetic particles, are brought together through magnetic forces.

MCTSs are 3D aggregates of tumor cells, alone or in combination with other cell types, generally grown in absence of exogenous scaffolds (9). However, the use of scaffolds has been also contemplated. MCTSs generally have a spherical geometry, possess a 3D architecture and an extracellular matrix (ECM) in which the cells are dispersed (10). Spheroids with diameter larger than 400–500 μm have usually a structure consisting of an exterior layer of proliferating cells, an intermediated layer of quiescent cells and an internal necrotic core (Figure 1A) (5). As a result, the protein and gene expression profiles of cancer cells in spheroids are more similar to those of tumor cells *in vivo*, allowing more reliable evaluations of anticancer drug effectiveness when administered as a solution or loaded in nanoparticle-based carriers (11, 12). MCTSs may be maintained in culture for 2–4 weeks, thus allowing long-term experiments (13). In addition, to better mimic the tumor microenvironment (cell-cell interactions), hybrid cell spheroids, also known as mixed-cell spheroids, can be easily obtained by co-culturing cancer cells with fibroblasts, endothelial cells, immune cells, and other cell types relevant to the tumor environment (14–16).

### Preparation Techniques

MCTSs may be produced by different protocols but the core principle, based on the anchorage-independent methodology, remains the same. By providing conditions in which cell-cell adhesive forces are greater than cell-substrate adhesive forces, cancer cells aggregate to form spheroids instead of adhering to a substrate (17). Hanging drop and liquid overlay are the most commonly employed methods to produce MCTSs (Figures 1B,C), but other methods, including agitation-induced systems, microencapsulation, and magnetic levitation, have been proposed (Figures 1D–F).

#### Hanging Drop

A drop of cell suspension is deposited on a dish lid and, upon inversion, cells are forced by gravity to accumulate at the free liquid–air interface and to form a single MCTS within the droplet (Figure 1B). This is the simplest and cheapest method to obtain MCTSs and co-cultures (mixed spheroids) since the use of dedicated materials, such as coated plates, might be avoided, but it requires MCTS transfer for further investigation. This method is very useful for generating uniform spheroids with a designated size and shape, even though long-term culture and media replacement remain a challenge (18). To overcome these limitations, companies have developed and commercialized different plates, such as Akura™ PLUS and Perfecta3D®, that simplify the culture procedures (19, 20).

#### Liquid Overlay

MCTSs can be easily generated by the liquid overlay, reducing interaction between the cells and the culture surface and forcing cell aggregation (Figure 1C). Culture plates treated for low attachment or suspension culture are now widely available on the market. Otherwise, standard plates can be easily coated with non-adhesive substrates such as poly-hydroxyhethylmethacrylate (Poly-HEMA) (21), agar (22), or agarose (23). The major limit of this technique is the formation of MCTSs with a high variability in sizes and shapes. To overcome this limit, it is possible to apply U-bottom 96-wells plate where a single spheroid per well is obtained. Alternatively, researchers can use *ad hoc* developed plates with multiple microcavities that allow the formation of one aggregate per cavity, allowing the production of a considerable number of consistent size spheroids [examples are AggreWell™ Microwell Plates (24, 25), Corning® Elplasia® Plates (26) and Sphericalplate 5D (27)].

#### Agitation-Induced Systems

Non-adherent conditions can be obtained in rotating systems, including gyratory shakers, perfusion bioreactors and spinner flasks [e.g., NASA bioreactor (28)] (Figure 1D). In these systems, the cell suspension is maintained in motion, preventing cell interaction with the bioreactor surface. While agitation-induced systems enabled production of large pools of MCTSs, the obtained spheroids are heterogeneous in size, shape and number of cell populations. For this reason, spheroid selection is required if the spheroid size needs to be controlled (29). Even though spheroid generation via bioreactors requires expensive instruments and high quantity of culture medium, bioreactors still provide greater advantages, mainly at the industrial level due to their scalability (29).

#### Microencapsulation

Taking advantage of the knowledge gained in pancreatic islet encapsulation (30, 31), cancer cell microencapsulation in alginate-poly-l-lysine-alginate beads has been proposed as a method to produce MCTSs (Figure 1E) (32). This method has the advantage to be scalable and to allow a precise control of the spheroid size and shape. However, microcapsules with a core-shell structure, seem to be more suitable for cell growing and MCTS production. Indeed, this structure makes it possible to use different materials with diverse chemical and physical characteristics for the core and the shell. For instance, Sakai et al. developed core-shell microcapsules by first embedding the tumor cells in gelatine beads and then surrounding them by calcium alginate membranes. The gelatine core liquefies upon incubation at 37°C allowing the formation of MCTSs that can be freed from the calcium alginate membranes by incubation with sodium citrate or alginate lyase (33, 34). The core-shell particles make it possible to study cancer cell lines unable to form MCTS by the techniques described above. However, the alginate membrane generally reduces oxygen and nutrient supply, the contact between cells, and may introduce a bias. To address these issues, Alessandri et al. developed a simple microfluidics platform to produce permeable and elastic hollow microspheres. The permeability of the gel allows easy diffusion of nutrients into the capsule and cell proliferation in a scaffold free environment (35).

#### Magnetic Levitation

Magnetic levitation was first developed by Souza et al. in 2010 (36). This system involves mixing the cells with magnetic particles and exposing them to a magnetic force during the culturing process (Figure 1F). It utilizes negative magnetophoresis, which mimics a weightless state. The magnetic force exerted causes the cells combined with magnetic particles to stay levitated or floating against gravity. This condition causes a geometry change of cell mass and stimulates cellular contact, leading to cell aggregation. In addition, this system can also facilitate multi-cellular co-culturing with different cell types (37–39). However, this technique presents a few drawbacks: beads are expensive and can be toxic to cells at a high concentration, and a limited number of aggregates can be produced (40).

## MCTSs in Nanomedicine Research

### Switching From 2D to 3D *in vitro* Models

The use of MCTSs is a great opportunity in cancer nanomedicine research since it can provide more reliable information than that obtained using 2D cell cultures. However, the differences between a spheroid and a solid tumor are still enormous and the results obtained should be interpreted with caution. In fact, the size of a MCTS is at least one order of magnitude smaller than the tumor mass and the effects observed *in vitro* could hardly be reproduced *in vivo*. However, the differences in size might be mitigated somehow by the fact that, *in vivo*, NPs reach the tumor through the systemic circulation by blood vessels/capillaries, taking advantage of the EPR effect (41). The distance between vessels and the tumor necrotic area *in vivo* has been reported to be 50–250 μm, compatible with the distance between the proliferating cell layer and the necrotic core in MCTSs (Figure 1A) (8).

When switching from 2D to 3D models, the data established in 2D models on NP penetration, drug release and anticancer effect will be difficult to compare with that obtained in the 3D models. For instance, the IC_50_ of an established anticancer drug can increase 1 log or more when moving from 2D to 3D, enhancing the concentrations necessary to have the effect (42). This can be even worst in the case of drug loaded NPs where NPs do not have to be taken up by a single cell layer, but must diffuse through multiple cell layers and the interstitial space.

**Figure 2:**
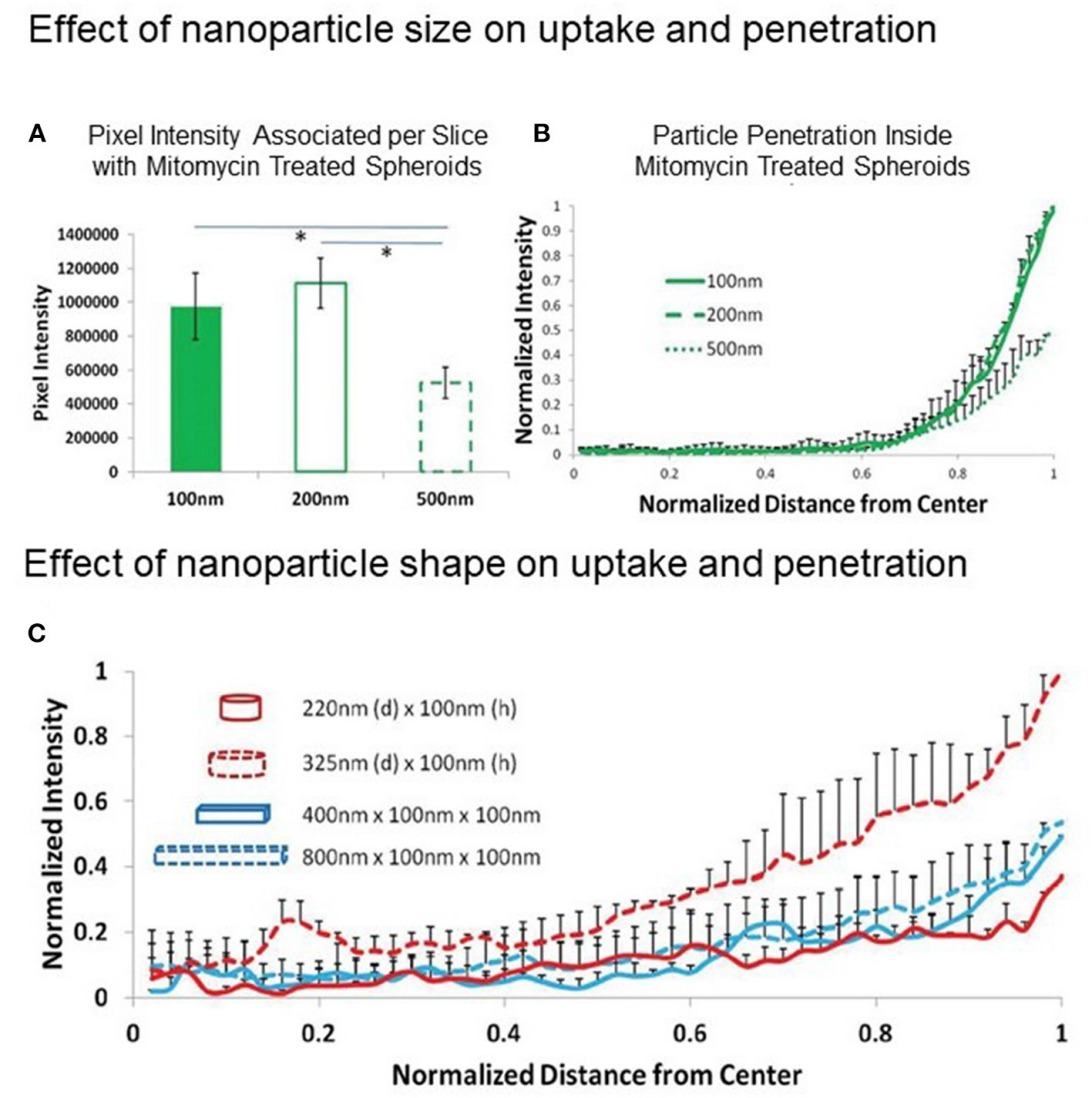
Effect of NP size and shape on MCTS uptake and penetration. **(A,B)** Effect of NP size: uptake and penetration of spherical 100, 200, and 500 nm polystyrene particles in Mitomycin C-treated HEK 293 spheroids. **(A)** Graph showing comparison of normalized pixel intensity associated with spheroid per slice with different diameter polystyrene beads and **(B)** Normalized radial intensity distribution of polystyrene beads as a function of distance from the center of the spheroid. **(C)** Effect of NP shape: normalized radial intensity distribution of shape-specific nanoparticles as a function of distance from the center of the spheroid. Only positive error bars are shown, to allow proper visualization of data. Reproduced with permission from Agarwal et al. (43) (John Wiley and Sons License, license number 5306710208408).

Obviously, one must consider the drug or NP diffusion process from the medium to the spheroid. By considering a diffusion process that obeys Fick's laws (normal diffusion), the different surface-to-volume ratios of the two model systems must be taken into account. MCTSs have much lower surface-to-volume ratios than cell culture monolayers and only the peripheral cell layer is directly exposed to the drug/NP that will diffuse according to the concentration gradient. NPs are 1-2 orders of magnitude larger than a small molecule and are expected to have a lower diffusion coefficient, diffusing very slowly from the spheroid surface to the center, with the risk of remaining confined in the outer cell layers. Therefore, particle shape, size and aspect ratio are features that affect the rate of NP diffusion through MCTSs, determining the deepness of penetration and the time needed to reach the center of the cell aggregate (Figure 2) (43). Also, the experiment timeframe in 2D and 3D models is generally different, as longer times are needed in 3D model experiments to record the effect expected from the treatment (42).

Obviously, NP penetration and distribution within MCTSs will never be just the result of normal diffusion. It is difficult to draw general considerations, given the different processes/mechanisms involved, such as NP cell uptake by endocytosis (pinocytosis and phagocytosis), NP intracellular trafficking, NP transcytosis, NP diffusion in the ECM.

By considering the not yet completely disclosed complexity of the two interacting systems (NP and MCTS), considerations on a case-by-case basis might be the best option. Indeed, “canonical” NP features, such as size, shape, surface chemistry/charge, surface decoration, are routinely measured and somehow controlled while others, such as the number of targeting moieties/nm^2^ and the adsorption of biomolecules (biomolecular corona), are often neglected or underestimated.

On the other hand, MCTS characteristics, such as cell line, preparation method, mean size/size distribution, cellularity, ECM secreted, and the eventual presence of exogenous ECM (i.e., hydrogels), will play a crucial role in the NP/spheroid interaction and the effect observed (6, 7, 44). Also, MCTS comprehensive morphological and molecular characterization will be essential in order to give a correct interpretation of the results obtained and to assess the model reproducibility.

#### MCTSs and Medium/High-Throughput Screening

The success of MCTSs as a pre-clinical model in anticancer drug development will also depend on the possibility to use them in medium-throughput screening (MTS) or high-throughput screening (HTS) (45, 46). This possibility will also open great opportunities in nanomedicine research, a field pioneered by Prof. Langer about 20 years ago (47), which has never been really developed. MTS or HTS assays for NPs will have a tremendous benefit for nanomedicine, especially now that nanoparticle-based mRNA therapeutics have been well recognized in clinics (48, 49). Thus, the use of 3D culture technologies, MCTSs *in primis*, in cancer nanomedicine MTS/HTS is a compelling unmet need.

From the 3D model side, three significant technical challenges hamper the development of these technologies: liquid handling automation, culture optimization and assay variability, and automated imaging/visualization of the 3D structure.

The automation of liquid handling can be conducted in suspension cultures, through the use of ultra-low-attachment microplates or the hanging drop technique (50). However, the application of automated liquid handling translates poorly when MCTSs are embedded in commercial thermogelling hydrogels. The use of thermogelling materials requires a highly controlled working environments and rapid processing due to their temperature-sensitive gelation conditions (51). Additionally, the batch-to-batch variability observed with some hydrogels considerably impacts the assay quality and reproducibility, crucial to ensure consistency when conducting MTS/HTS (52). Finally, MCTSs produced by co-culturing multiple cell types have much greater morphological complexity than simpler spheroids and 2D cultures, and multiparametric analysis will be required to investigate and quantify the cell response the treatment (53).

In the case of nanomedicine, an accurate analysis of nanoparticle uptake and/or localization would be of great value. However, their tridimensionality also poses a difficulty in computational image analysis and visualization: the complex topology and the thickness of MCTSs, as for other 3D models, make image analysis challenging, and are incompatible with most automated imaging systems due to low light penetration and absorption across the multi-layered structures (52).

Despite these challenging barriers, new culture platforms and imaging systems are being developed to overcome the technical issues that hinder the use of MCTSs in MTS/HTS. These developments include using synthetic hydrogels to generate more consistent 3D cell cultures, automated high-resolution imaging using light-sheet microscopy, and integrated computational platforms for data analysis and visualization of 3D structure (54–56).

However, from a nanomaterial perspective, the issue of producing and handling NP libraries remain unsolved. In fact, MTS/HTS can be relatively easily applicable to the material *per se* (polymers, lipids) but it become a struggle in the case of complex NPs designed and engineered for cancer drug delivery and targeting. Nanotechnology-based medicines under scrutiny today, given their surface functionalization with fragile biomacromolecules and their anticancer drug cargo, are much more difficult to handle than small molecule drugs.

## Conclusions and Perspective

The application of MCTSs in cancer nanomedicine offers a great opportunity, since it is the first step to narrow the gap between pre-clinical and clinical research, making the process of nanotechnology-based medicine development more efficient and successful. More important, this will surely pave the way for the routine application of more advanced *in vitro* 3D models, such as organ-on-a-chip and organoids, in academia and industry. The use of MCTS in MTS/HTS is still challenging but recent technological advances might solve some of the issues, making this possible in the near future.

However, the switch from 2D to 3D *in vitro* models will not be painless, since researchers will be obliged to consider the higher complexity of the model during the design of the experiment, for example, adapting the experiment timeframe and the treatment concentrations as well as the assays needed to have the desired outcome. It will be necessary to go deeper in MCTS biological characterization (e.g., cell and molecular biology, biochemistry) to know and, if possible, control the experimental parameters that will affect the experimental results.

In addition, since most, if not all, *in vitro* data available from the last 40 years of nanotechnology-based drug delivery research were generated on classical 2D models, for which comparisons with 3D models will be extremely difficult, researchers will have to contemplate a sort of “re-evaluation” of NP technologies discarded because of their failure on cell culture monolayer assays.

In conclusion, MCTSs will become the standard *in vitro* model to assess the nanomedicines performance in adulthood cancers, and cell culture monolayers will be used to obtain complementary information, such as mechanistic understandings of NP uptake, binding affinity, and translocation into intracellular compartments.

## Data Availability Statement

The original contributions presented in the study are included in the article, further inquiries can be directed to the corresponding author.

## Author Contributions

MR and PB conceptualized the idea, wrote the manuscript, made the figures, reviewed, and approved the final version of the manuscript. Both authors contributed to the article and approved the submitted version.

## Funding

MR's fellowship was financed by the Department of Pharmacy and Biotechnology (FaBiT) of the University of Bologna (Project *Sviluppo di organoidi di osteosarcoma per lo screening di nuovi chemioterapici*).

## Conflict of Interest

The authors declare that the research was conducted in the absence of any commercial or financial relationships that could be construed as a potential conflict of interest.

## Publisher's Note

All claims expressed in this article are solely those of the authors and do not necessarily represent those of their affiliated organizations, or those of the publisher, the editors and the reviewers. Any product that may be evaluated in this article, or claim that may be made by its manufacturer, is not guaranteed or endorsed by the publisher.

## References

[1] Bissell MJ. Goodbye flat biology - time for the 3rd and the 4th dimensions. J Cell Sci. (2017) 130:3–5. 10.1242/jcs.200550 28043963

[2] Van Zundert I Fortuni B Rocha S. From 2D to 3D cancer cell models-the enigmas of drug delivery research. Nanomaterials (Basel). (2020) 10:236. 10.3390/nano10112236 33187231 PMC7696259

[3] Rodrigues J Heinrich MA Teixeira LM Prakash J. 3D *in vitro* model (r)evolution: unveiling tumor-stroma interactions. Trends Cancer. (2021) 7:249–64. 10.1016/j.trecan.2020.10.009 33218948

[4] Mehta G Hsiao AY Ingram M Luker GD Takayama S. Opportunities and challenges for use of tumor spheroids as models to test drug delivery and efficacy. J Control Release. (2012) 164:192–204. 10.1016/j.jconrel.2012.04.045 22613880 PMC3436947

[5] Gebhard C Gabriel C Walter I. Morphological and immunohistochemical characterization of canine osteosarcoma spheroid cell cultures. Anat Histol Embryol. (2016) 45:219–30. 10.1111/ahe.12190 26287450 PMC4949528

[6] Leong DT Ng KW. Probing the relevance of 3D cancer models in nanomedicine research. Adv Drug Deliv Rev. (2014) 79–80:95–106. 10.1016/j.addr.2014.06.007 24996135

[7] Lazzari S Couvreur P Mura S. Multicellular tumor spheroids: a relevant 3D model for the *in vitro* preclinical investigation of polymer nanomedicines. Polym Chem. (2017) 8:4947–69. 10.1039/C7PY00559H

[8] Lu H Stenzel MH. Multicellular tumor spheroids (MCTS) as a 3D *in vitro* evaluation tool of nanoparticles. Small. (2018) 14:e1702858. 10.1002/smll.201702858 29450963

[9] Friedrich J Ebner R Kunz-Schughart LA. Experimental anti-tumor therapy in 3-D: spheroids–old hat or new challenge? Int J Radiat Biol. (2007) 83:849–71. 10.1080/09553000701727531 18058370

[10] LaBarbera DV Reid BG Yoo BH. The multicellular tumor spheroid model for high-throughput cancer drug discovery. Expert Opin Drug Discov. (2012) 7:819–30. 10.1517/17460441.2012.708334 22788761

[11] Katt ME Placone AL Wong AD Xu ZS Searson PC. *In vitro* tumor models: advantages, disadvantages, variables, and selecting the right platform. Front Bioeng Biotechnol. (2016) 4:12. 10.3389/fbioe.2016.00012 26904541 PMC4751256

[12] Goodman TT Ng CP Pun SH. 3-D tissue culture systems for the evaluation and optimization of nanoparticle-based drug carriers. Bioconjug Chem. (2008) 19:1951–9. 10.1021/bc800233a 18788773 PMC2652657

[13] Hsiao AY Tung YC Kuo CH Mosadegh B Bedenis R Pienta KJ. Micro-ring structures stabilize microdroplets to enable long term spheroid culture in 384 hanging drop array plates. Biomed Microdev. (2012) 14:313–23. 10.1007/s10544-011-9608-5 22057945 PMC3304008

[14] Lamichhane SP Arya N Kohler E Xiang S Christensen J Shastri P. Recapitulating epithelial tumor microenvironment *in vitro* using three dimensional tri-culture of human epithelial, endothelial, and mesenchymal cells. BMC Cancer. (2016) 16:581. 10.1186/s12885-016-2634-1 27484993 PMC4971675

[15] Matte I Legault CM Garde-Granger P Laplante C Bessette P Rancourt C. Mesothelial cells interact with tumor cells for the formation of ovarian cancer multicellular spheroids in peritoneal effusions. Clin Exp Metastasis. (2016) 33:839–52. 10.1007/s10585-016-9821-y 27612856

[16] Ho VH Guo WM Huang CL Ho SF Chaw SY Tan EY. Manipulating magnetic 3D spheroids in hanging drops for applications in tissue engineering and drug screening. Adv Healthc Mater. (2013) 2:1430–4. 10.1002/adhm.201200408 23606526

[17] Weiswald LB Bellet D Dangles-Marie V. Spherical cancer models in tumor biology. Neoplasia. (2015) 17:1–15. 10.1016/j.neo.2014.12.004 25622895 PMC4309685

[18] Kelm JM Timmins NE Brown CJ Fussenegger M Nielsen LK. Method for generation of homogeneous multicellular tumor spheroids applicable to a wide variety of cell types. Biotechnol Bioeng. (2003) 83:173–80. 10.1002/bit.10655 12768623

[19] Hofmann S Cohen-Harazi R Maizels Y Koman I. Patient-derived tumor spheroid cultures as a promising tool to assist personalized therapeutic decisions in breast cancer. Transl Cancer Res. (2022) 11:134–47. 10.21037/tcr-21-1577 35261891 PMC8841497

[20] Lee G Kim H Park JY Kim G Han J Chung S. Generation of uniform liver spheroids from human pluripotent stem cells for imaging-based drug toxicity analysis. Biomaterials. (2021) 269:120529. 10.1016/j.biomaterials.2020.120529 33257114

[21] Dangles V Femenia F Laine V Berthelemy M Le Rhun D Poupon MF. Two- and three-dimensional cell structures govern epidermal growth factor survival function in human bladder carcinoma cell lines. Cancer Res. (1997) 57:3360–4. 9269996

[22] Yuhas JM Li AP Martinez AO Ladman AJ. A simplified method for production and growth of multicellular tumor spheroids. Cancer Res. (1977) 37:3639–43. 908012

[23] Dangles-Marie V Pocard M Richon S Weiswald LB Assayag F Saulnier P. Establishment of human colon cancer cell lines from fresh tumors versus xenografts: comparison of success rate and cell line features. Cancer Res. (2007) 67:398–407. 10.1158/0008-5472.CAN-06-0594 17210723

[24] Razian G Yu Y Ungrin M. Production of large numbers of size-controlled tumor spheroids using microwell plates. J Vis Exp. (2013) 81:50665. 10.3791/50665 24300192 PMC3991351

[25] Antonchuk J. Formation of embryoid bodies from human pluripotent stem cells using AggreWell plates. Methods Mol Biol. (2013) 946:523–33. 10.1007/978-1-62703-128-8_32 23179853

[26] Limraksasin P Okawa H Zhang M Kondo T Osathanon T Pavasant P. Size-optimized microspace culture facilitates differentiation of mouse induced pluripotent stem cells into osteoid-rich bone constructs. Stem Cells Int. (2020) 2020:7082679. 10.1155/2020/7082679 32508932 PMC7244985

[27] Wassmer CH Lebreton F Bellofatto K Perez L Cottet-Dumoulin D Andres A. Bio-engineering of pre-vascularized islet organoids for the treatment of type 1 diabetes. Transpl Int. (2021) 35:10214. 10.3389/ti.2021.10214 35185372 PMC8842259

[28] Lei XH Ning LN Cao YJ Liu S Zhang SB Qiu ZF. NASA-approved rotary bioreactor enhances proliferation of human epidermal stem cells and supports formation of 3D epidermis-like structure. PLoS ONE. (2011) 6:e26603. 10.1371/journal.pone.0026603 22096490 PMC3212516

[29] Breslin S O'Driscoll L. Three-dimensional cell culture: the missing link in drug discovery. Drug Discov Today. (2013) 18:240–9. 10.1016/j.drudis.2012.10.003 23073387

[30] Orive G Hernandez RM Rodriguez Gascon A Calafiore R Chang TM de Vos P. History, challenges and perspectives of cell microencapsulation. Trends Biotechnol. (2004) 22:87–92. 10.1016/j.tibtech.2003.11.004 14757043

[31] Giovagnoli S Luca G Blasi P Mancuso F Schoubben A Arato I. Alginates in Pharmaceutics and Biomedicine: Is the Future so Bright? Curr Pharm Des. (2015) 21:4917–35. 10.2174/1381612821666150820105639 26290204

[32] Zhang X Wang W Yu W Xie Y Zhang X Zhang Y. Development of an *in vitro* multicellular tumor spheroid model using microencapsulation and its application in anticancer drug screening and testing. Biotechnol Prog. (2005) 21:1289–96. 10.1021/bp050003l 16080713

[33] Sakai S Ito S Kawakami K. Calcium alginate microcapsules with spherical liquid cores templated by gelatin microparticles for mass production of multicellular spheroids. Acta Biomater. (2010) 6:3132–7. 10.1016/j.actbio.2010.02.003 20144915

[34] Sakai S Inamoto K Liu Y Tanaka S Arii S Taya M. Multicellular tumor spheroid formation in duplex microcapsules for analysis of chemosensitivity. Cancer Sci. (2012) 103:549–54. 10.1111/j.1349-7006.2011.02187.x 22168771 PMC7713610

[35] Alessandri K Sarangi BR Gurchenkov VV Sinha B Kiessling TR Fetler L. Cellular capsules as a tool for multicellular spheroid production and for investigating the mechanics of tumor progression *in vitro*. Proc Natl Acad Sci U S A. (2013) 110:14843–8. 10.1073/pnas.1309482110 23980147 PMC3773746

[36] Souza GR Molina JR Raphael RM Ozawa MG Stark DJ Levin CS. Three-dimensional tissue culture based on magnetic cell levitation. Nat Nanotechnol. (2010) 5:291–6. 10.1038/nnano.2010.23 20228788 PMC4487889

[37] Anil-Inevi M Yaman S Yildiz AA Mese G Yalcin-Ozuysal O Tekin HC. Biofabrication of *in situ* self assembled 3D cell cultures in a weightlessness environment generated using magnetic levitation. Sci Rep. (2018) 8:7239. 10.1038/s41598-018-25718-9 29740095 PMC5940762

[38] Kim JA Choi JH Kim M Rhee WJ Son B Jung HK. High-throughput generation of spheroids using magnetic nanoparticles for three-dimensional cell culture. Biomaterials. (2013) 34:8555–63. 10.1016/j.biomaterials.2013.07.056 23937911

[39] Lewis NS Lewis EE Mullin M Wheadon H Dalby MJ Berry C. Magnetically levitated mesenchymal stem cell spheroids cultured with a collagen gel maintain phenotype and quiescence. J Tissue Eng. (2017) 8:2041731417704428. 10.1177/2041731417704428 28616152 PMC5460809

[40] Hoarau-Vechot J Rafii A Touboul C Pasquier J. Halfway between 2D and animal models: are 3D cultures the ideal tool to study cancer-microenvironment interactions? Int J Mol Sci. (2018) 19:181. 10.3390/ijms19010181 29346265 PMC5796130

[41] Bertrand N Wu J Xu X Kamaly N Farokhzad OC. Cancer nanotechnology: the impact of passive and active targeting in the era of modern cancer biology. Adv Drug Deliv Rev. (2014) 66:2–25. 10.1016/j.addr.2013.11.009 24270007 PMC4219254

[42] Berrouet C Dorilas N Rejniak KA Tuncer N. Comparison of drug inhibitory effects (IC_50_) in monolayer and spheroid cultures. Bull Math Biol. (2020) 82:68. 10.1007/s11538-020-00746-7 32495209 PMC9773863

[43] Agarwal R Jurney P Raythatha M Singh V Sreenivasan SV Shi L. Effect of shape, size, and aspect ratio on nanoparticle penetration and distribution inside solid tissues using 3D spheroid models. Adv Healthc Mater. (2015) 4:2269–80. 10.1002/adhm.201500441 26376024 PMC9346573

[44] Han SJ Kwon S Kim KS. Challenges of applying multicellular tumor spheroids in preclinical phase. Cancer Cell Int. (2021) 21:152. 10.1186/s12935-021-01853-8 33663530 PMC7934264

[45] Ryan SL Baird AM Vaz G Urquhart AJ Senge M Richard DJ. Drug discovery approaches utilizing three-dimensional cell culture assay. Drug Dev Technol. (2016) 14:19–28. 10.1089/adt.2015.670 26866750

[46] Montanez-Sauri SI Beebe DJ Sung KE. Microscale screening systems for 3D cellular microenvironments: platforms, advances, and challenges. Cell Mol Life Sci. (2015) 72:237–49. 10.1007/s00018-014-1738-5 25274061 PMC4629475

[47] Lynn DM Anderson DG Putnam D Langer R. Accelerated discovery of synthetic transfection vectors: parallel synthesis and screening of a degradable polymer library. J Am Chem Soc. (2001) 123:8155–6. 10.1021/ja016288p 11506588

[48] Rui Y Wilson DR Tzeng SY Yamagata HM Sudhakar D Conge M. High-throughput and high-content bioassay enables tuning of polyester nanoparticles for cellular uptake, endosomal escape, and systemic *in vivo* delivery of mRNA. Sci Adv. (2022) 8:eabk2855. 10.1126/sciadv.abk2855 34985952 PMC8730632

[49] Tome I Francisco V Fernandes H Ferreira L. High-throughput screening of nanoparticles in drug delivery. APL Bioeng. (2021) 5:031511. 10.1063/5.0057204 34476328 PMC8397474

[50] Langhans SA. Three-dimensional *in vitro* cell culture models in drug discovery and drug repositioning. Front Pharmacol. (2018) 9:6. 10.3389/fphar.2018.00006 29410625 PMC5787088

[51] Ruel-Gariepy E Leroux JC. *In situ*-forming hydrogels–review of temperature-sensitive systems. Eur J Pharm Biopharm. (2004) 58:409–26. 10.1016/j.ejpb.2004.03.019 15296964

[52] Booij TH Price LS Danen EHJ. 3D cell-based assays for drug screens: challenges in imaging, image analysis, and high-content analysis. SLAS Discov. (2019) 24:615–27. 10.1177/2472555219830087 30817892 PMC6589915

[53] Horvath P Aulner N Bickle M Davies AM Nery ED Ebner D. Screening out irrelevant cell-based models of disease. Nat Rev Drug Discov. (2016) 15:751–69. 10.1038/nrd.2016.175 27616293

[54] Worthington P Drake KM Li Z Napper AD Pochan DJ Langhans. Beta-hairpin hydrogels as scaffolds for high-throughput drug discovery in three-dimensional cell culture. Anal Biochem. (2017) 535:25–34. 10.1016/j.ab.2017.07.024 28757092 PMC5576559

[55] Eismann B Krieger TG Beneke J Bulkescher R Adam L Erfle H. Automated 3D light-sheet screening with high spatiotemporal resolution reveals mitotic phenotypes. J Cell Sci. (2020) 133:43. 10.1242/jcs.245043 32295847 PMC7286290

[56] Bilgin CC Fontenay G Cheng Q Chang H Han J Parvin B. BioSig3D: high content screening of three-dimensional cell culture models. PLoS ONE. (2016) 11:e0148379. 10.1371/journal.pone.0148379 26978075 PMC4792475

